# Dietary caprylic acid, *Campylobacter jejuni* colonisation and product quality in broilers: a systematic review and meta-analysis

**DOI:** 10.5194/aab-69-477-2026

**Published:** 2026-09-02

**Authors:** Muhammet Kuddusi Erhan

**Affiliations:** 1 Department of Crop and Animal Production, Vocational High School, Ağrı İbrahim Çeçen University, Ağrı, Türkiye

## Abstract

This systematic review and meta-analysis re-evaluated how dietary caprylic acid (CA) supplementation affects *Campylobacter jejuni* colonisation and growth performance in broiler chickens, and which factors modulate its efficacy. Because caecal colonisation at the end of rearing is a principal determinant of carcass contamination at processing, and because growth performance governs slaughter weight and carcass yield, the two outcomes were treated here not as independent endpoints but as complementary dimensions of a single theme: the microbiological and technological quality of the final poultry product. We searched PubMed, Scopus and Google Scholar for controlled in vivo broiler trials published between January 2000 and July 2025 that administered CA orally and reported quantitative outcomes for caecal *C. jejuni* counts or growth performance. Data were extracted from the primary publications and synthesised with random-effects models, using the mean difference (MD, log10 colony-forming units (CFU) g^−1^) for colonisation and the standardised mean difference (SMD) for growth, with pre-planned subgroup, dose–response and sensitivity analyses. Twelve studies met the eligibility criteria; 11 contributed 15 comparisons to the quantitative syntheses and one could be included only qualitatively. Caprylic acid reduced caecal *C. jejuni* by a pooled -1.46 log10 CFU g^−1^ (95 % confidence interval (CI) -2.50 to -0.41 ; P=0.006 ), with substantial heterogeneity ( $I^{2}=88.1$ %). Route of administration accounted for most of that heterogeneity: in-feed application reduced colonisation by -2.68 log10 CFU g^−1^ (95 % CI -3.88 to -1.49 ; $I^{2}=39.0$ %), whereas water-based application had no detectable effect ( -0.05 log10 CFU g^−1^; 95 % CI -0.68 to +0.58 ). Growth performance was unaffected overall (SMD +0.16 ; 95 % CI -0.32 to +0.64 ; P=0.524 ), but the dose–response analysis showed that inclusion above about 1 % of feed depressed feed intake and body weight while conferring no additional antimicrobial benefit. Funnel-plot asymmetry was significant (Egger's test, P=0.009 ) but coincided with the feed-versus-water contrast, so publication bias and genuine subgroup heterogeneity could not be separated. Caprylic acid is therefore a credible component of antibiotic-free *Campylobacter* control in broilers when delivered in feed at approximately 0.35 % to 0.875 % but not when delivered in drinking water.

## 1 Introduction

*Campylobacter* spp., and particularly *Campylobacter jejuni*, are major bacterial agents of food-borne gastroenteritis worldwide, and impose a substantial public health and economic burden in both industrialised and developing settings (Kaakoush et al., 2015). Poultry meat and derived products are the dominant source of human exposure through the direct consumption of undercooked products and through cross-contamination during food handling (Sahin et al., 2015; EFSA and ECDC, 2023). In commercial broiler production, flock-level colonisation rates with *C. jejuni* commonly range from about 55 % to 100 %, so the broiler intestinal tract acts as a major reservoir (Allen et al., 2011; Sahin et al., 2015). At slaughter, leakage of caecal contents or crop spillage contaminates carcass surfaces and amplifies the risk along the food chain (Hermans et al., 2011; Sahin et al., 2015).

Poultry production long relied on antimicrobial drugs, initially as growth-promoting feed additives and in parallel as therapeutic tools. The global spread of antimicrobial-resistant bacteria made that practice increasingly untenable (Castanon, 2007), and antibiotic growth promoters are now banned in the European Union and severely restricted or voluntarily abandoned in many other markets (Castanon, 2007; EFSA and ECDC, 2023). Consumer demand for antibiotic-free poultry has intensified in parallel, obliging the industry to invest in alternative strategies for gut health and pathogen reduction (EFSA and ECDC, 2023; Van Deun et al., 2008; Ricke, 2003). Researchers have consequently examined a wide range of natural or nature-identical feed additives, including phytogenic compounds, probiotics, prebiotics, organic acids and essential oils (Ricke, 2003; Meunier et al., 2016).

Among these alternatives, caprylic acid (octanoic acid) has attracted particular attention. This eight-carbon medium-chain fatty acid occurs naturally in coconut and palm kernel oils, and is bactericidal against *C. jejuni* in vitro at low millimolar concentrations (Hermans et al., 2010). Its amphipathic, lipophilic character allows it to insert into and disrupt bacterial membranes, interfere with the electron transport chain, lower intracellular pH and inhibit key metabolic enzymes (Ricke, 2003; Desbois and Smith, 2010). In broilers, dietary inclusion at around 0.7 % reduced gastrointestinal *C. jejuni* loads by approximately three log10 colony-forming units (CFU) g^−1^ (Solís de los Santos et al., 2008, 2009), which suggested that the compound might serve as a practical pre-harvest intervention.

The evidence is not consistent, however. Reported effect sizes and durations of action vary markedly with dose, route of administration, bird age and health status, and experimental design. In-feed experiments generally report clear reductions in caecal colonisation (Solís de los Santos et al., 2008, 2010), whereas water-based applications give less uniform results, with some trials showing transient reductions or none at all (Metcalf et al., 2011; Hermans et al., 2012). The effect on performance traits is equally ambiguous. Some studies report that medium-chain fatty acid (MCFA) mixtures improve body-weight gain or feed efficiency (van Gerwe et al., 2010; Lin et al., 2022), others find neutral effects at moderate doses (Kollanoor-Johny et al., 2012) and still others report growth depression at inclusion rates of 1.4 % (Solís de los Santos et al., 2008).

Framed thematically, the two outcomes examined in this review are two facets of poultry product quality rather than unrelated endpoints. Caecal *C. jejuni* load at the end of the rearing period is the strongest single predictor of carcass and meat contamination during processing, and therefore of the microbiological quality, regulatory compliance and shelf life of the retail product (Hermans et al., 2011; EFSA and ECDC, 2023). Growth performance, in turn, determines slaughter weight, carcass yield and flock uniformity, which are the principal technological and economic quality attributes of broiler meat. An additive that lowers pathogen load at the cost of impaired growth, or that improves growth without any food-safety benefit, has limited practical value. This synthesis therefore evaluates caprylic acid explicitly as a product-quality intervention, in which antimicrobial efficacy and zootechnical performance are assessed jointly rather than in isolation.

This heterogeneity makes it difficult to draw firm conclusions about the overall efficacy and optimal use of caprylic acid in broiler production. Characterising the magnitude of the effect, its sources of variability and its practical implications requires an approach that combines qualitative synthesis with quantitative pooling. The present work therefore reports a systematic review and meta-analysis of in vivo broiler studies in which caprylic acid was administered orally and *C. jejuni* colonisation or growth performance was measured, together with subgroup, dose–response, sensitivity and publication-bias analyses that examine how dose, formulation and route of administration shape the observed outcomes.

## 2 Materials and methods

### 2.1 Study protocol

We conducted this systematic review and meta-analysis to summarise and quantify the effects of caprylic acid on *C. jejuni* colonisation and growth performance in broiler chickens (*Gallus gallus domesticus*). We prepared the protocol in accordance with the PRISMA 2020 recommendations and followed those guidelines throughout (Page et al., 2021).

### 2.2 Literature search strategy

We searched PubMed, Scopus and Google Scholar for articles published between 1 January 2000 and 30 July 2025. The search strategy combined three concepts: intervention, target population and outcomes. Intervention terms were “caprylic acid”, “octanoic acid”, “medium-chain fatty acid”, “MCFA” and “monocaprylin”; population terms were “broiler”, “chicken” and “poultry”; outcome terms were “Campylobacter”, “*Campylobacter jejuni*”, “growth performance”, “body weight gain” and “feed conversion ratio”. We combined these with Boolean operators and adapted each search string to the syntax of the database concerned. We also screened the reference lists of key primary studies and relevant reviews by hand to capture eligible articles that the database queries might have missed.

### 2.3 Eligibility criteria and study selection

Two reviewers screened all retrieved records independently, in two stages. In the first stage, they assessed titles and abstracts, and removed clearly irrelevant items. In the second, they examined the full texts of potentially eligible articles against predefined criteria. We included a study if it was a peer-reviewed original research article; if the experiment used broiler chickens; if caprylic acid or a caprylic-acid-containing mixture was administered orally in feed or drinking water; if at least one concurrent control group was present; and if it reported quantitative outcomes for caecal *C. jejuni* counts, for growth performance or for both. We excluded in vitro experiments, reviews, conference abstracts and theses; studies evaluating only post-harvest interventions such as carcass decontamination; studies without an appropriate control group or without sufficient quantitative data; and studies in poultry species other than broiler chickens. We also excluded studies in which caprylic acid was given only as an unspecified component of a multi-ingredient additive, because the exposure cannot then be quantified and the comparison cannot be assigned to a dose or formulation stratum.

The reviewers resolved disagreements at any stage by discussion and, where necessary, by consulting a third researcher. We documented the final set of included studies and the reasons for exclusion in accordance with the PRISMA flow diagram (Page et al., 2021).

### 2.4 Data extraction and management

Two reviewers extracted data from each eligible study independently, using a standardised form. We recorded general study characteristics (first author, year, country), experimental design (bird strain, number of animals per group, age at the start of the intervention, trial duration), intervention characteristics (form and dose of caprylic acid, duration and route of administration, whether the compound was given alone or in an MCFA blend and whether the intent was prophylactic or therapeutic) and outcome measures. For colonisation, we recorded mean caecal *C. jejuni* counts (log10 CFU g^−1^) with their dispersion measures, and sample sizes for control and treatment groups. For growth performance, we recorded final body weight, body-weight gain and average daily gain where available. Three points about the extracted data require explicit statement. First, dispersion measures were recorded as reported and converted only where the reporting convention was unambiguous. Where a study reported the standard error of the mean, we obtained the standard deviation as SD = SE × √n ; and where a study reported a pooled standard error from an analysis of variance, we treated it as applying to every group in that analysis. One study reported standard deviations rather than standard errors (Hermans et al., 2012) and was handled accordingly. Second, where a study presented outcome data only in figures, we read the values from the published figure and have flagged those comparisons as figure derived. Third, where a study reported outcomes only qualitatively or stated that data were not shown, the comparison could not enter the quantitative synthesis and is discussed narratively instead. The two extractors reconciled all discrepancies before the dataset was finalised. Study-level characteristics and outcome data are summarised in Tables 1 and 2.

### 2.5 Statistical analysis

For colonisation, we used the mean difference (MD) in log10 CFU g^−1^ between treatment and control groups as the effect measure. For growth performance, which the primary studies expressed on different scales, we used the standardised mean difference with the Hedges' correction for small samples (Hedges' g ). We report every effect size with its 95 % confidence interval (CI). Because differences in study design and intervention protocol made considerable heterogeneity likely, we applied random-effects models throughout. All analyses were re-run for this revision from the re-extracted dataset using a single reproducible script, which is available from the corresponding author.

Between-study heterogeneity was quantified by Cochran's Q statistic, by the between-study variance $\tau^{2}$ estimated with the DerSimonian–Laird method and by the $I^{2}$ statistic; $I^{2}$ values of approximately 25 %, 50 % and 75 % were taken to indicate low, moderate and substantial heterogeneity, respectively (DerSimonian and Laird, 1986; Higgins et al., 2003; Page et al., 2021). Differences between subgroups were tested formally using the Q statistic for subgroup differences ( Q _between) on k-1 degrees of freedom, rather than being inferred from the overlap of subgroup confidence intervals. We pre-specified subgroup analyses to explore the sources of heterogeneity. For colonisation, we grouped comparisons by route of administration (feed versus drinking water) and by formulation (pure caprylic acid versus MCFA mixtures). For growth performance, we grouped them by dose level (low to moderate versus high) and by whether caprylic acid was given alone or in a mixture with other medium-chain fatty acids or with *Yucca schidigera* extract. Two subgroupings that we had originally planned, by purpose of use (prophylactic versus therapeutic) and by challenge status of the birds, could not be carried out because the resulting strata contained too few comparisons to support a stable estimate.

Three unit-of-analysis situations arose and were handled as follows. First, where a study reported two or more fully independent experiments, each with its own control group, we entered them as separate comparisons; this applies to the two replicate trials of Metcalf et al. (2011), which had separate controls and gave discordant results. Second, where a study compared several treatment arms against a single shared control group, entering each arm as an independent comparison would count that control group more than once. This applies to the 0.7 % and 1.4 % growth performance arms of Solís de los Santos et al. (2008). We therefore divided the control group among the arms sharing it, following the approach recommended for multi-arm trials, and we report below the effect of this correction. Third, where a study reported a graded dose series against one control, we did not pool the arms at all; they are presented descriptively in the dose–response analysis (Sect. 3.4), where the correlation between the resulting estimates is stated explicitly. We also carried out sensitivity analyses. In the leave-one-out analysis, each comparison was removed in turn, so that the influence of any single dataset on the pooled estimate could be assessed.

The funnel plot illustrates the relationship between effect size and study precision for comparisons examining the impact of caprylic acid on caecal *C. jejuni* counts. Each point represents an individual comparison, plotted by its mean difference in log10 CFU g^−1^ on the horizontal axis and its standard error on the vertical axis. The vertical line marks the overall pooled effect estimate. The asymmetric distribution of points, with an apparent paucity of small studies reporting neutral or unfavourable results, together with the statistically significant Egger regression test, suggests the presence of small-study effects and possible publication bias.

## 3 Results

### 3.1 Characteristics of included studies

The search and selection process identified 12 original studies that evaluated caprylic acid or caprylic-acid-containing mixtures in broiler chickens between 2008 and 2022 (Solís de los Santos et al., 2008, 2009, 2010; van Gerwe et al., 2010; Kollanoor Johny et al., 2009; Kollanoor-Johny et al., 2012; Metcalf et al., 2011; Hejdysz et al., 2012; Hermans et al., 2012; Begum et al., 2015; Hovorková and Skřivanová, 2015; and Lin et al., 2022). Eleven of these contributed 15 comparisons to the quantitative syntheses: six to the colonisation analysis and nine to the growth analysis. Hovorková and Skřivanová (2015) reported no numerical caecal counts, and this study is therefore discussed narratively only; and van Gerwe et al. (2010) reported colonisation as a CD50 threshold, which is not commensurate with the log10 CFU g^−1^ scale and contributes to the growth analysis alone. One further study identified at the full-text stage, Gracia et al. (2016), was excluded under this criterion: it screened 12 feed additives, among them a medium-chain fatty acid preparation and a monoglyceride blend, but does not report the caprylic acid content or dose of either preparation, therefore its comparisons could not be placed on the dose–formulation scale used here. Summary characteristics are given in Tables 1 and 2. Full bibliographic details of the 12 included studies are given in Appendix A.

**Table 1:** Effects of caprylic acid supplementation on *Campylobacter jejuni* colonisation in broiler chickens.

Study and year	Treatment group (dose, duration, route)	Control (mean log10 CFU g^−1^ ± SE)	Treatment (mean log10 CFU g^−1^ ± SE)	n (animals/group)	p value	Notes
Solís de los Santos et al. (2008)	0.7 % CA, 10 d, feed	8.3±0.2	6.3±0.5	10	<0.05	Prophylactic, 10 d old chicks
Solís de los Santos et al. (2009)	0.7 % CA, 3 d, feed	∼7.4	∼4.8	10	<0.05	Therapeutic, 42 d old birds, no withdrawal
Solís de los Santos et al. (2010)	0.7 % CA, 3 d, feed	∼6.8	∼3.5	10	<0.05	Therapeutic, 42 d old birds, no withdrawal
Metcalf et al. (2011) (Trial 1)	0.175 % CA, 3 d, water	8.0±0.3	5.1±1.2	10	<0.05	Water-soluble form, effective
Metcalf et al. (2011) (Trial 2)	0.175 % CA, 3 d, water	7.9±0.1	8.1±0.2	10	>0.05	Ineffective result
Hermans et al. (2012)	0.4 % MCFA, 3 d, water	∼7.1	∼7.2	12	>0.05	MCFA emulsion, ineffective
van Gerwe et al. (2010)	1 % MCFA, continuous, feed	CD50 = 2.5 log CFU	CD50 = 4.8 log CFU	87/227	<0.05	Treatment group more resistant to colonisation
Hovorková and Skřivanová (2015)	0.5 % CA, continuous, feed	No significant difference	No significant difference	48 (total)	>0.05	No caecal difference, transient faecal reduction

The table summarises the design and key features of the in vivo broiler experiments that reported caecal *C. jejuni* outcomes. For each study, it gives the first author and year, bird strain, age at the start of the intervention, housing and challenge conditions, treatment details (dose, duration, route and formulation), control group characteristics, sample size per group and the main colonisation outcomes (mean log10 CFU g^−1^ with the reported dispersion measure). The notes state whether the intervention was prophylactic or therapeutic and flag methodological features that bear on interpretation.

**Table 2:** Effects of caprylic acid supplementation on growth performance in broiler chickens.

Study and year	Treatment group (dose, duration, route)	Control (BWG/BW ± SE)	Treatment (BWG/BW ± SE)	n (animals/replicate)	p value	Notes
Kollanoor Johny et al. (2009)	0.7 % CA, 18 d, feed	850.3±32.2 g	841.1±17.2 g	6	>0.05	Final BW; Salmonella challenge
Kollanoor-Johny et al. (2012)	0.7 % CA, 6 weeks, feed	2860±60 g	2800±50 g	14	≥0.05	Final BW; Salmonella challenge
Solís de los Santos et al. (2008)	0.7 % CA, 10 d, feed	206.8±7.7 g	196.2±8.6 g	10	>0.05	Final BW; *C. jejuni* challenge
Solís de los Santos et al. (2008)	1.4 % CA, 10 d, feed	206.8±7.7 g	179.1±1.1 g	10	<0.05	High dose reduced BW
Solís de los Santos et al. (2009)	0.7 % CA, 42 d, feed (last 3–7 d)	No significant difference	No significant difference	10	>0.05	Final BW
Solís de los Santos et al. (2010)	0.7 % CA, 42 d, feed (last 3 d)	2850.0±151.0 g	3007.2±117.8 g	10	>0.05	Final BW
Begum et al. (2015)	100 mg kg^−1^ CA + Yucca, 35 d, feed	1529 g	1692 g	14	<0.05	BWG; combination effect
van Gerwe et al. (2010)	1 % MCFA, >14 d, feed	–	+49±24 g	87/227	0.044	BWG increase; MCFA positive effect
Lin et al. (2022)	3000/1000 g t^−1^ MMG, 42 d, feed	54.36±0.578 g	58.08±0.578 g	8	0.028	ADG increase; MCFA mixture
Hejdysz et al. (2012)	0.85 % CA, 35 d, feed	2232 g	2270 g	12	0.0613	BWG; non-significant trend

Abbreviations: BW: body weight; BWG: body-weight gain; ADG: average daily gain; MMG: monoglyceride mixture.

The table lists all broiler studies that reported growth performance in relation to caprylic acid supplementation. For each comparison, it gives the first author and year, bird strain, experimental duration, dose and route, formulation type (pure CA or MCFA blend), the outcome variable assessed and the main statistical result. The comments state whether the birds were experimentally challenged; whether the growth response was neutral, beneficial or detrimental; and which dose ranges impaired performance.

Doses ranged from 0.04375 % to 1.4 % of feed or water, and intervention durations from 3 d courses to continuous administration throughout a production cycle. Most experiments used young broilers between 10 d and 6 weeks of age. The compound was delivered in feed or in drinking water, as pure caprylic acid or within an MCFA blend, and in both prophylactic and therapeutic settings (Solís de los Santos et al., 2008, 2009, 2010; Metcalf et al., 2011; Hermans et al., 2012; van Gerwe et al., 2010).

### 3.2 Effect of caprylic acid on *Campylobacter jejuni* colonisation

**Figure 1:**
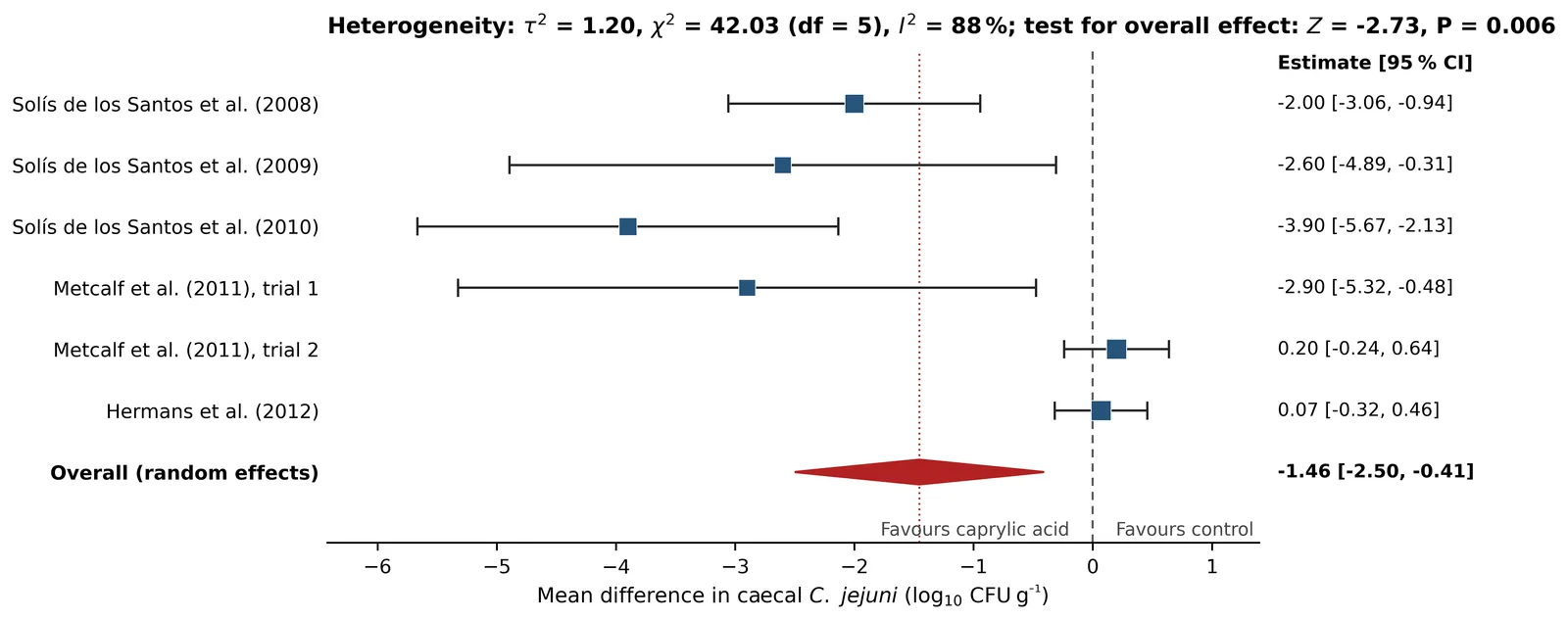
Forest plot of the pooled effect of caprylic acid supplementation on caecal *Campylobacter jejuni* colonisation in broiler chickens. Squares are the mean difference for each comparison, sized in proportion to the random-effects weight; horizontal lines are 95 % confidence intervals; and the diamond is the pooled estimate. Negative values indicate lower caecal loads under caprylic acid. Heterogeneity statistics appear in the panel header.

Six comparisons from five studies reported caecal *C. jejuni* counts on a common log10 CFU g^−1^ scale. Caprylic acid supplementation reduced caecal *C. jejuni* by a pooled mean difference of -1.46 log10 CFU g^−1^ (95 % CI -2.50 to -0.41 ; Z=2.73 , P=0.006 ), and the confidence interval did not cross the line of no effect (Fig. 1). Between-study heterogeneity was substantial ( $\tau^{2}=1.20$ ; Q=42.03 , df = 5, P<0.001 ; $I^{2}=88.1$ %). The pooled value should therefore be read as the average of genuinely different underlying effects rather than as an estimate of a single common effect.

**Figure 2:**
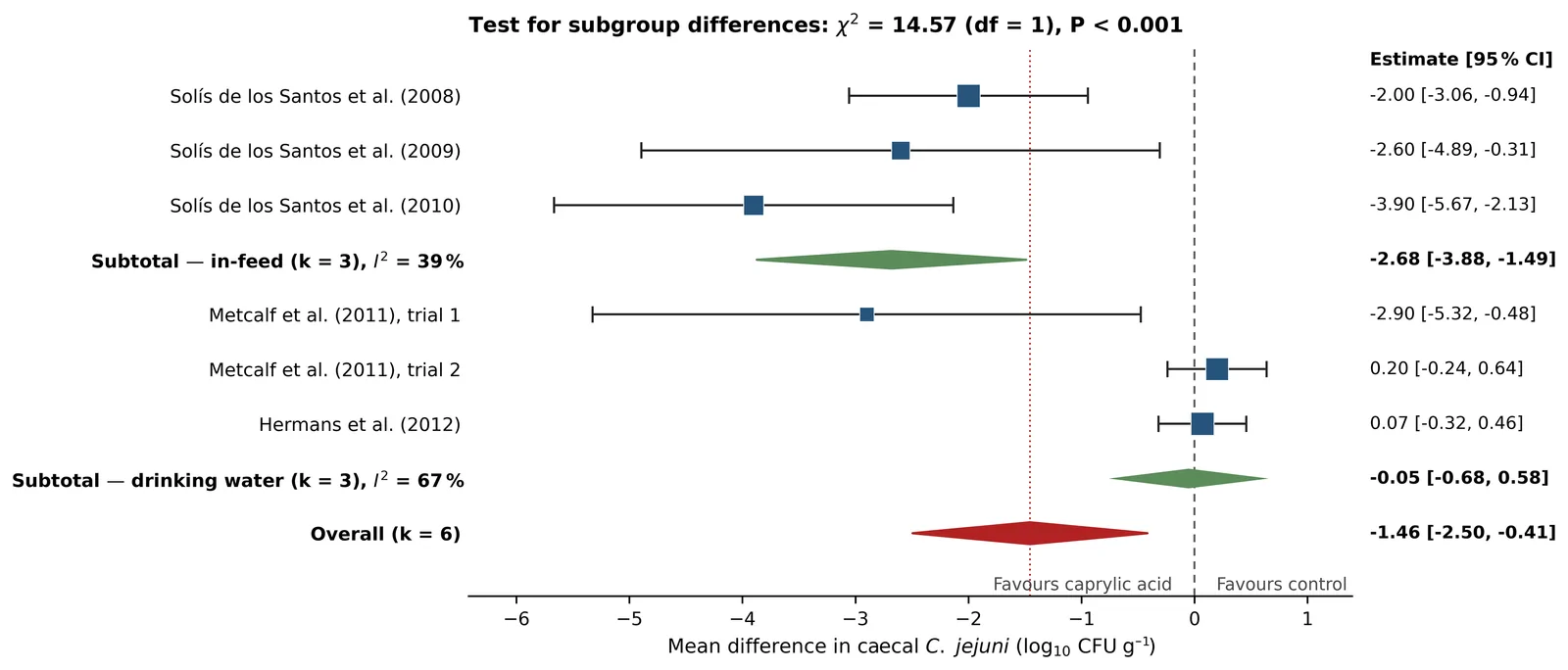
Subgroup forest plot of the effect of caprylic acid on *Campylobacter jejuni* colonisation according to route of administration. Comparisons are stratified by route of administration. Green diamonds are subgroup estimates, and the red diamond is the overall estimate; subgroup $I^{2}$ values are given in the row labels and the test for subgroup differences in the panel header.

The pre-specified subgroup analysis by route of administration accounted for most of this inconsistency (Fig. 2). Across the three in-feed comparisons, the pooled reduction was -2.68 log10 CFU g^−1^ (95 % CI -3.88 to -1.49 ; Z=4.40 , P<0.001 ), with only moderate residual heterogeneity ( $I^{2}=39.0$ %). Across the three drinking-water comparisons, there was essentially no effect ( -0.05 log10 CFU g^−1^; 95 % CI -0.68 to +0.58 ; Z=0.16 , P=0.876 ), and these trials remained heterogeneous among themselves ( $I^{2}=67.2$ %). The formal test for subgroup differences was significant ( Q _between = 14.57, df = 1, P<0.001 ). The fall in $I^{2}$ from 88.1 % overall to 39.0 % within the in-feed subgroup identifies the delivery matrix as the principal source of between-study inconsistency for this outcome.

Within the in-feed subgroup, 0.7 % caprylic acid given for 3 to 10 d reduced caecal counts by 2.0 to 3.9 log10 CFU g^−1^ in three independent experiments spanning both prophylactic use in 10 d old chicks and therapeutic use in market-aged birds (Solís de los Santos et al., 2008, 2009, 2010). By contrast, water-soluble caprylic acid produced a large reduction in one trial but none in a replicate of the same experiment (Metcalf et al., 2011), and a medium-chain fatty acid emulsion delivered in drinking water left caecal counts unchanged (Hermans et al., 2012). Dietary caprylic acid at 0.25 to 0.5 % likewise reduced faecal shedding only transiently, with no difference in caecal counts at slaughter (Hovorková and Skřivanová, 2015). These patterns are summarised in Table 3.

### 3.3 Effect of caprylic acid on growth performance

**Figure 3:**
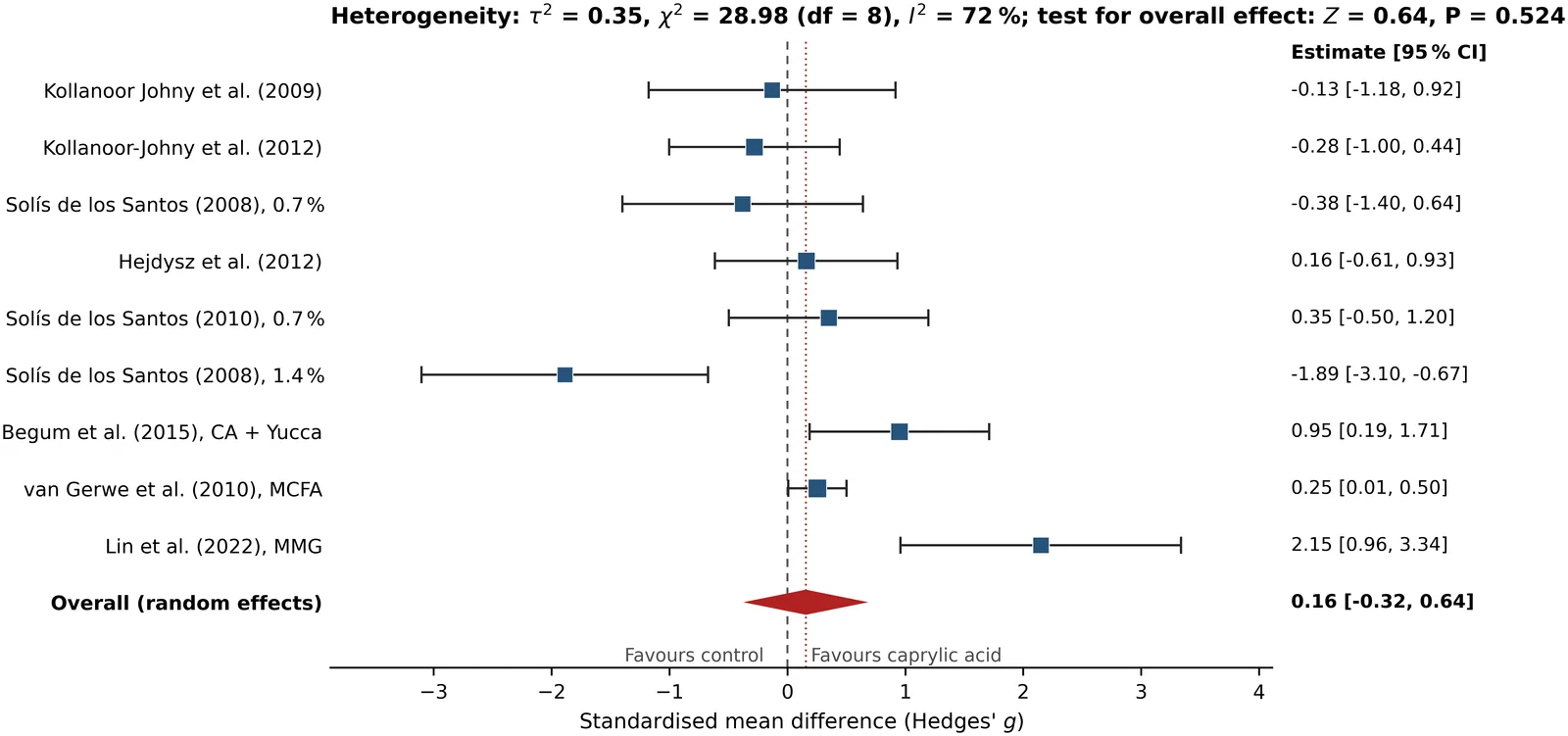
Forest plot of the overall effect of caprylic acid supplementation on growth performance in broiler chickens. Squares are the standardised mean difference (Hedges' g ) for each comparison, sized in proportion to the random-effects weight; the diamond is the pooled estimate. Positive values favour caprylic acid. Heterogeneity statistics appear in the panel header.

**Figure 4:**
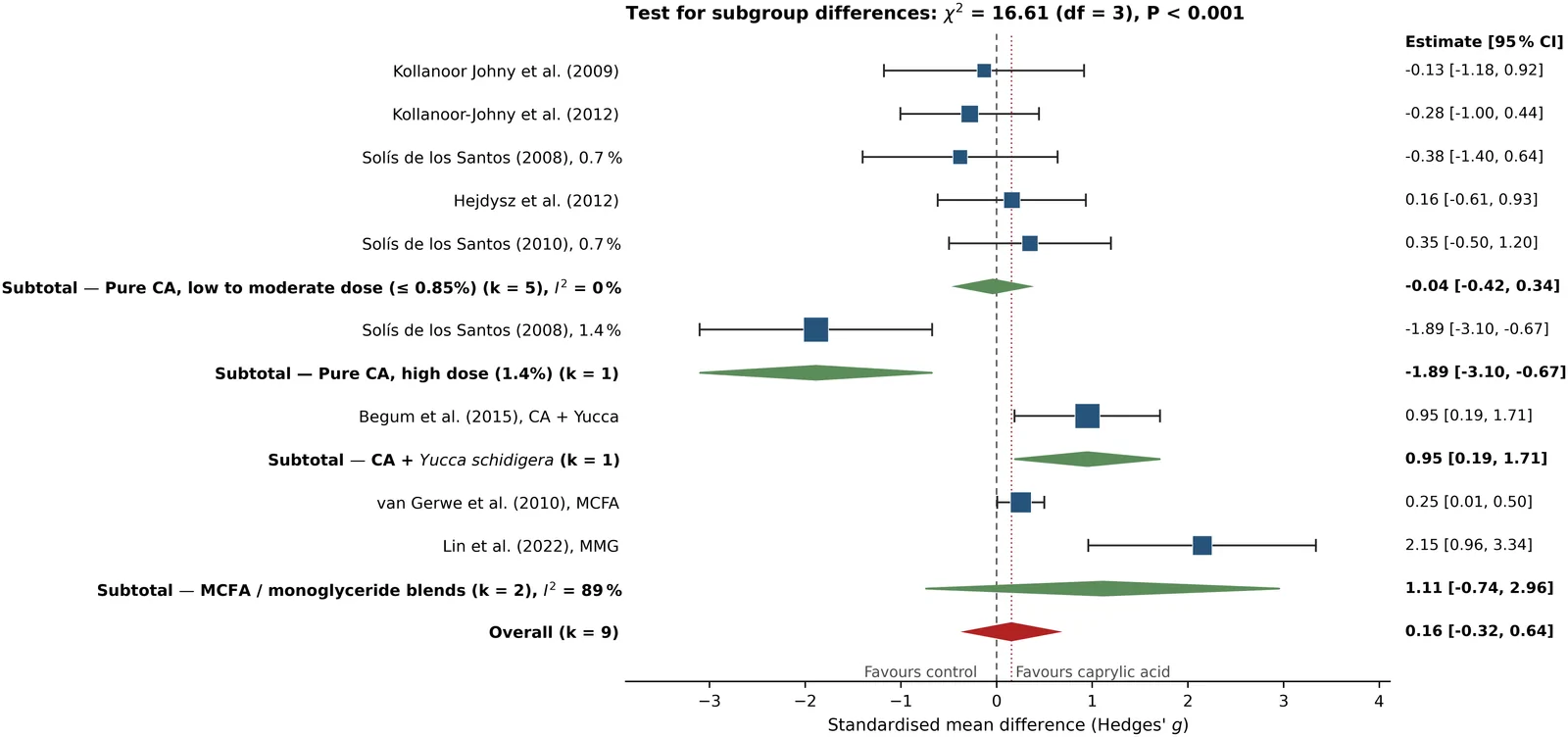
Subgroup forest plot of growth performance outcomes stratified by caprylic acid dose and formulation. Comparisons are stratified by dose and formulation. Green diamonds are subgroup estimates, and the red diamond is the overall estimate; the test for subgroup differences appears in the panel header. The two blend subgroups each rest on comparisons in which the contribution of caprylic acid cannot be isolated (Sect. 3.3).

Nine comparisons from eight studies reported growth performance as final body weight, body-weight gain or average daily gain. The pooled standardised mean difference was +0.16 (95 % CI -0.32 to +0.64 ; Z=0.64 , P=0.524 ), indicating no overall effect of caprylic acid on growth (Fig. 3). Heterogeneity was again substantial ( $\tau^{2}=0.35$ ; Q=28.98 , df = 8, P<0.001 ; $I^{2}=72.4$ %). Dividing the shared control group of Solís de los Santos et al. (2008) between its two treatment arms, rather than counting it twice, changed the pooled estimate from +0.14 to +0.16 and $I^{2}$ from 73.9 % to 72.4 %; the corrected values are reported throughout. As for colonisation, this inconsistency was structured rather than random: it arose almost entirely from differences between intervention types, which are examined below and illustrated in Fig. 4.

The subgroup analysis nevertheless revealed a clear structure (Fig. 4). Pure caprylic acid at low to moderate doses left growth unchanged: the pooled estimate for these five comparisons was -0.04 (95 % CI -0.42 to +0.34 ; P=0.843 ), with no detectable heterogeneity ( $I^{2}=0.0$ %). By contrast, at 1.4 % of feed, caprylic acid significantly reduced body weight ( g=-1.89 ; 95 % CI -3.10 to -0.67 ) (Solís de los Santos et al., 2008). Formulations combining caprylic acid with *Yucca schidigera* extract ( g=+0.95 ; 95 % CI +0.19 to +1.71 ) (Begum et al., 2015) or with other medium-chain fatty acids and monoglycerides ( g=+1.11 ; 95 % CI -0.74 to +2.96 ) (van Gerwe et al., 2010; Lin et al., 2022) pointed in the opposite direction, although the latter subgroup was itself markedly heterogeneous ( $I^{2}=89.3$ %). The test for subgroup differences was significant ( Q _between = 16.61, df = 3, P<0.001 ). Two cautions apply to the blend subgroups: in Begum et al. (2015), the caprylic acid dose was only 100 mg kg^−1^ (0.01 % of feed), and the effect cannot be separated from that of *Yucca schidigera*; and in Lin et al. (2022), the reported P value refers to the omnibus comparison across six treatment groups, with the relevant pairwise contrast non-significant by the authors' own post-hoc test. Neither comparison therefore supports a claim that caprylic acid itself improves growth. These patterns are detailed in Table 4.

**Table 3:** Summary of *Campylobacter jejuni* colonisation outcomes by route, dose and formulation of caprylic acid.

Subgroup	Number of comparisons	Dose range (caprylic acid / MCFA)	Route of administration	Formulation type	Pooled MD, log10 CFU g^−1^ (95 % CI)	$I^{2}$ and P value	Key notes
Feed, pure CA (prophylactic and therapeutic)	3	0.7 % CA (3–10 d)	Feed	Pure caprylic acid	-2.68 ( -3.88 to -1.49 )	$I^{2}=39.0$ %; Z=4.40 , P<0.001	Prophylactic or short therapeutic courses; no withdrawal period before slaughter. Solís de los Santos trials and related experiments [7,8,17]
Feed, pure CA, continuous (not pooled)	1	0.5 % CA (continuous)	Feed	Pure caprylic acid	Not estimable – no numerical caecal data reported	P>0.05 as reported by the authors	Continuous use; only a transient reduction in faecal shedding was reported
Feed, MCFA blend (different outcome scale)	1	1 % MCFA mixture (continuous)	Feed	MCFA blend	Not poolable – CD50 threshold, not log10 CFU g^−1^	P<0.05 as reported by the authors	CD50 shifted from 2.5 to 4.8 log CFU in treatment group; interpreted as improved protection against colonisation
Water, pure CA (short 3 d courses)	2	0.175 % CA (3 d)	Drinking water	Water-soluble caprylic acid	-2.90 ( -5.32 to -0.48 ) and +0.20 ( -0.24 to +0.64 )	Replicate trials discordant	Short 3 d courses; one trial showed robust reduction; another found no meaningful difference
Water, MCFA emulsion	1	0.4 % MCFA (3 d)	Drinking water	MCFA emulsion	+0.07 ( -0.32 to +0.46 )	P=0.72	Water-based emulsion; final caecal colonisation remained similar between groups

Abbreviations: CA: caprylic acid; MCFA: medium-chain fatty acids; CD50: challenge dose required to colonise 50 % of the birds in a group.

Rows are ordered by route of administration and formulation. For each stratum, the table gives the number of contributing comparisons, the dose and duration, the pooled mean difference with its 95 % confidence interval, the corresponding $I^{2}$ and P value, and interpretive notes. Two strata could not be pooled: Hovorková and Skřivanová (2015) report no numerical caecal counts, and van Gerwe et al. (2010) report a CD50 threshold rather than a caecal count.

### 3.4 Dose–response relationship

**Figure 5:**
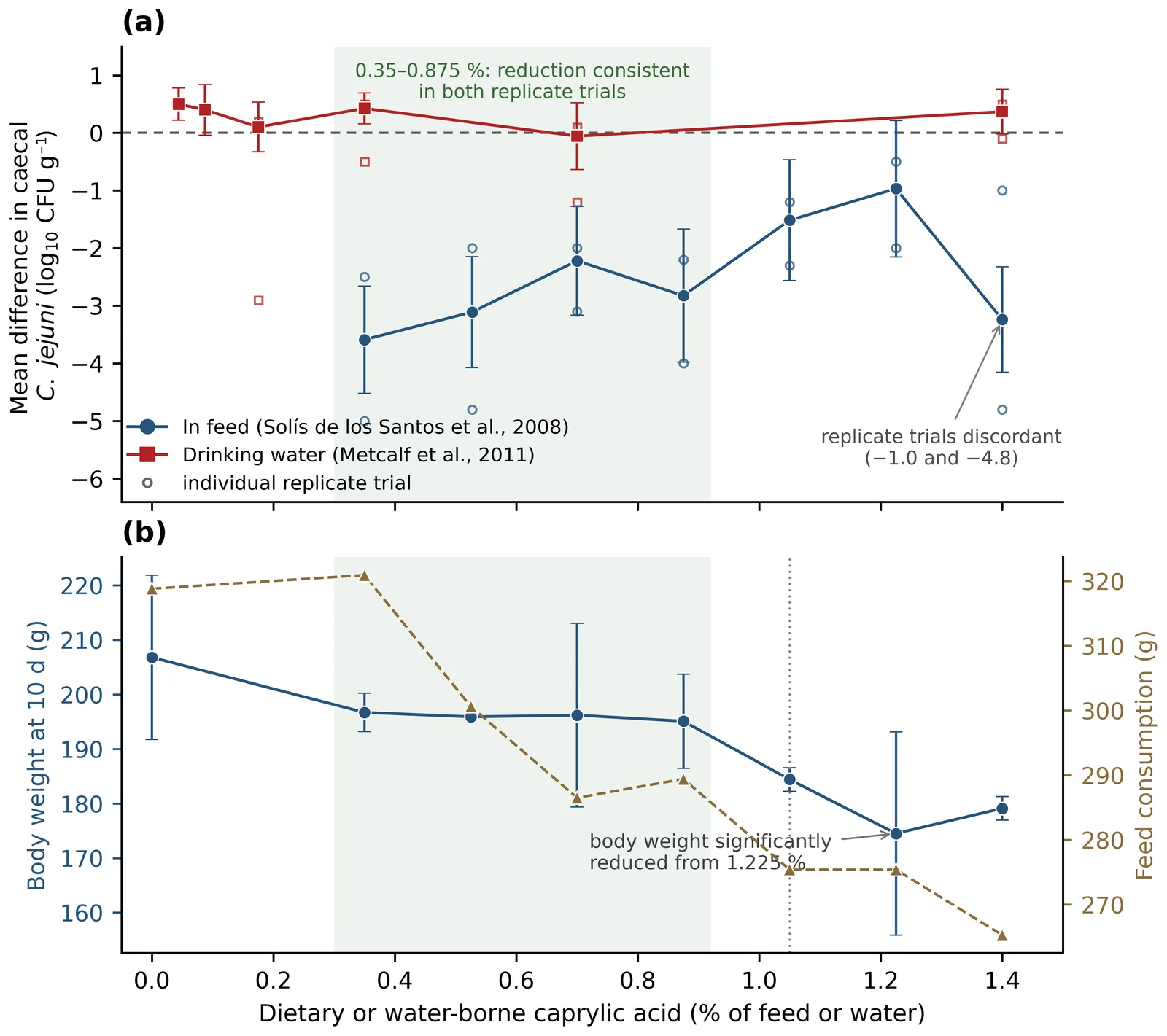
Dose–response relationship for caprylic acid in broiler chickens. **(a)** Mean difference in caecal *Campylobacter jejuni* counts by concentration for in-feed administration (Solís de los Santos et al., 2008) and administration in drinking water (Metcalf et al., 2011). Filled symbols are within-study fixed-effect estimates pooled across replicate trials with 95 % confidence intervals; open symbols are the individual replicate trials. The shaded band marks the concentration range over which a reduction was observed in both replicate trials. **(b)** Body weight and feed consumption at 10 d of age across the same in-feed dose series (Solís de los Santos et al., 2008). Estimates within a study share a common control group and are therefore correlated.

Two of the included studies tested a graded series of caprylic acid concentrations and therefore permit a dose–response description, although the small number of independent studies precludes formal meta-regression. Solís de los Santos et al. (2008) evaluated seven in-feed concentrations between 0.35 % and 1.4 % in two replicate trials, and Metcalf et al. (2011) evaluated six water-borne concentrations between 0.04375 % and 1.4 %, also in two replicate trials. Because each treatment arm within a trial was compared with a single shared control group, the resulting estimates are correlated and are presented descriptively rather than pooled (Fig. 5a).

In feed, concentrations between 0.35 % and 0.875 % produced a reduction in caecal *C. jejuni* in both replicate trials, with pooled within-study mean differences of -3.59 , -3.11 , -2.22 and -2.82 log10 CFU g^−1^ at 0.35 %, 0.527 %, 0.7 % and 0.875 %, respectively. Above this range, the estimated effect attenuated to -1.51 log10 CFU g^−1^ at 1.05 % and -0.97 log10 CFU g^−1^ at 1.225 %, and the two replicate trials became discordant: at 1.4 %, the reduction was -1.0 log10 CFU g^−1^ in one trial and -4.8 log10 CFU g^−1^ in the other. The relationship is therefore best described not as a monotonic dose–response but as an effective range of roughly 0.35 % to 0.875 %, above which the response becomes both weaker on average and markedly less reproducible.

The zootechnical data from the same experiment indicate why higher concentrations are not simply better (Fig. 5b). Feed consumption declined progressively across the dose series, from 318.8 g at 0 % to 265.3 g at 1.4 %, and body weight at 10 d was significantly reduced relative to control at 1.225 % and 1.4 % ( 174.5±9.5 and 179.1±1.1 g versus 206.8±7.7 g; P<0.05 ), whereas concentrations up to 0.875 % left body weight unchanged. Palatability therefore appears to impose an upper practical limit before any pharmacological ceiling is reached. It should be noted, however, that reduced intake alone does not fully explain the loss of antimicrobial efficacy, because total caprylic acid consumption still rose with dose (approximately 3.7 g at 1.4 % versus 2.0 g at 0.7 %); the mechanism underlying the plateau remains unresolved.

In drinking water, no dose–response was detectable at all. Across the six concentrations tested, estimated mean differences ranged from -0.06 to +0.50 log10 CFU g^−1^ with no ordering by dose, and the single substantial reduction observed at 0.175 % in the first trial ( -2.9 log10 CFU g^−1^) was not reproduced at the same concentration in the second trial ( +0.2 log10 CFU g^−1^). The absence of any concentration-dependent effect in water, over a 32-fold dose range, reinforces the conclusion drawn from the subgroup analysis that the delivery matrix, rather than the amount administered, is the primary determinant of efficacy.

### 3.5 Publication bias and sensitivity analyses

**Figure 6:**
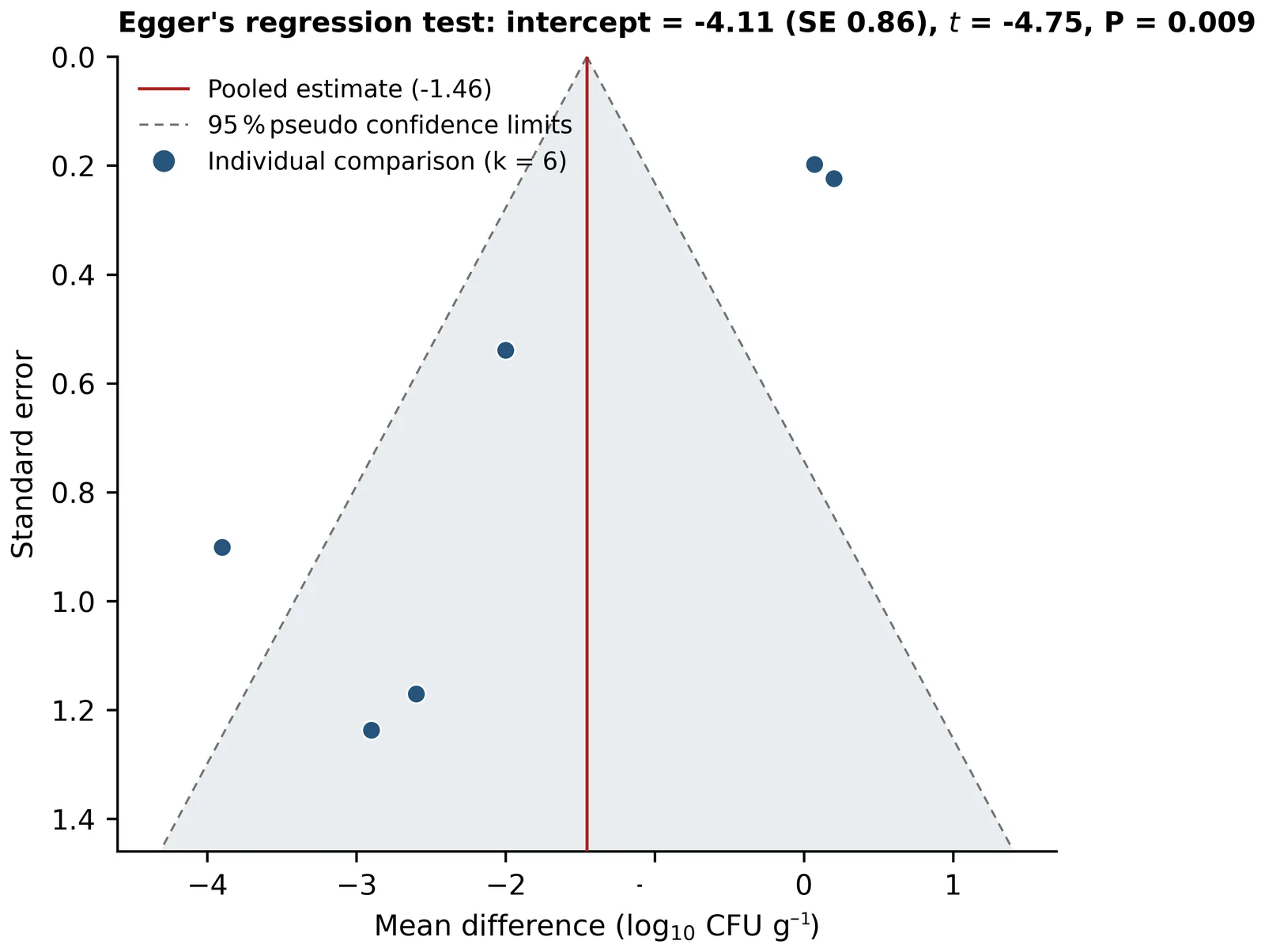
Funnel plot assessing potential publication bias in studies evaluating the effect of caprylic acid on *Campylobacter jejuni* colonisation.

**Figure 7:**
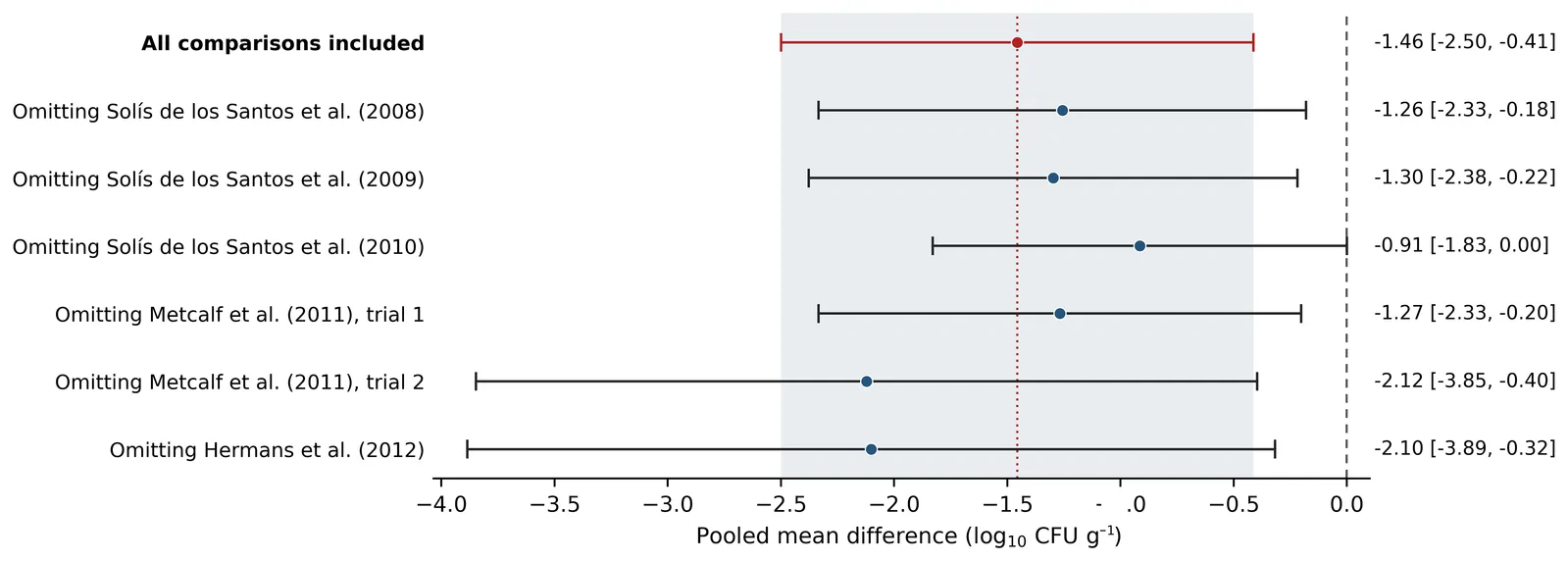
Leave-one-out sensitivity analysis for the effect of caprylic acid supplementation on caecal *Campylobacter jejuni* colonisation. Each row corresponds to an iteration in which one comparison was omitted and shows the recalculated pooled mean difference in caecal *C. jejuni* counts (log10 CFU g^−1^) with its 95 % confidence interval; the shaded band marks the confidence interval of the full model. The point estimates remain negative throughout, ranging from -0.91 to -2.12 log10 CFU g^−1^, so the direction of the effect does not depend on any single comparison. The confidence interval reaches zero in one iteration, when Solís de los Santos et al. (2010) is omitted, indicating that statistical significance is marginally dependent on that trial.

Visual inspection of the funnel plot for the colonisation outcome showed clear asymmetry (Fig. 6): the three least-precise comparisons all lay well to the left of the pooled estimate, whereas the two most-precise comparisons lay close to the line of no effect. Egger's regression test confirmed this impression (intercept = -4.11 , SE = 0.86; t=-4.75 , df = 4, P=0.009 ). Two qualifications are necessary. First, with only six comparisons the test has low power and is sensitive to individual data points, so its result should be treated as descriptive rather than confirmatory; for the same reason, no formal adjustment method such as trim and fill was applied, since these procedures are unreliable when fewer than 10 studies are available (Sterne et al., 2011). Second, the asymmetry maps precisely onto the route-of-administration subgroup: all three in-feed comparisons are comparatively imprecise and strongly negative, whereas the two most-precise comparisons are both water based and close to null. Funnel asymmetry of this kind can be generated by genuine between-subgroup heterogeneity as readily as by selective publication, and with six comparisons the two explanations cannot be distinguished. The leave-one-out analysis (Fig. 7) yielded a negative pooled estimate in every iteration, ranging from -0.91 to -2.12 log10 CFU g^−1^. The effect remained statistically significant in five of the six iterations; when Solís de los Santos et al. (2010) was omitted, the pooled estimate was - 0.91 log10 CFU g^−1^ (95 % CI -1.83 to 0.00), that is, exactly at the boundary of significance. The direction of the effect is therefore robust to the removal of any single comparison, whereas its statistical significance depends to a modest degree on the single largest trial. These results are summarised in Table 5.

**Table 4:** Summary of growth performance outcomes by dose and formulation of caprylic acid in broiler chickens.

Subgroup	Number of comparisons	Representative dose and duration	Pooled SMD, Hedges' g (95 % CI)	$I^{2}$ and P value	Key notes
Pure CA, low to moderate dose ( ≤0.85 % in feed)	5	0.7 %–0.85 % CA in feed (10 d to 6 weeks)	-0.04 ( -0.42 to +0.34 )	$I^{2}=0.0$ %; Z=0.20 , P=0.843	Final BW or BWG generally not significantly different from controls in Salmonella and *C. jejuni* challenge studies [3,7,8,10,14]. One trial reports a non-significant trend towards higher BWG at 0.85 % CA
Pure CA, high dose (1.4 % in feed)	1	1.4 % CA in feed (10 d)	-1.89 ( -3.10 to -0.67 )	Single comparison; P=0.002 ; shared control divided	High inclusion level reduced body weight in young broilers, suggesting a dose-dependent negative effect
CA + *Yucca schidigera* extract (CA-based blend)	1	100 mg kg^−1^ CA + Yucca (35 d, feed)	+0.95 ( +0.19 to +1.71 )	Single comparison; P=0.014	Combination of CA with *Yucca schidigera* extract has increased body-weight gain; interpreted as a synergistic formulation
MCFA blends including CA (MCFA mixtures / monoglycerides)	2	1 % MCFA in feed ( ≥14 d) or MMG mixtures (42 d)	+1.11 ( -0.74 to +2.96 )	$I^{2}=89.3$ %; Z=1.18 , P=0.240	MCFA mixtures, including caprylic acid or its monoglyceride forms, have improved growth performance in both BWG and ADG measures [5,11]

Abbreviations: BW: body weight; BWG: body-weight gain; ADG: average daily gain; MCFA: medium-chain fatty acids; MMG: monoglyceride mixture.

The table groups the growth performance comparisons by dose level and formulation: pure caprylic acid at low to moderate doses ( ≤0.85 % of feed), pure caprylic acid at high dose (1.4 % of feed), caprylic acid combined with *Yucca schidigera* extract, and MCFA or monoglyceride mixtures containing caprylic acid. For each subgroup, it gives the representative dose and duration, the number of contributing comparisons, the pooled standardised mean difference with its 95 % confidence interval and interpretive remarks. Moderate doses of pure caprylic acid left growth unchanged, the 1.4 % dose reduced body weight, and the blend subgroups point towards improvement but rest on comparisons in which the contribution of caprylic acid itself cannot be isolated.

**Table 5:** Summary of sensitivity and publication-bias analyses in the caprylic acid meta-analysis.

Outcome/analysis	Approach	Main quantitative findings	Interpretation
*C. jejuni* colonisation (mean difference in log10 CFU g^−1^)	Leave-one-out sensitivity analysis (random-effects model)	Pooled estimate negative in all six iterations, ranging from -0.91 to -2.12 log10 CFU g^−1^; statistically significant in five of six. Omitting Solís de los Santos et al. (2010) gives -0.91 (95 % CI -1.83 to 0.00)	The direction of the effect is robust to omission of any single comparison. Statistical significance is marginally dependent on the largest trial, which should be acknowledged rather than treated as full robustness.
*C. jejuni* colonisation (mean difference in log10 CFU g^−1^)	Funnel plot and Egger's regression test	Funnel plot markedly asymmetric (Fig. 6). Egger's test: intercept =-4.11 (SE 0.86), t=-4.75 , df = 4, P=0.009 . Trim and fill not applied ( k<10 )	Small-study effects are present, but the asymmetry coincides exactly with the feed-versus-water subgroup split, so selective publication and genuine subgroup heterogeneity cannot be distinguished at k=6 . The pooled magnitude may be overestimated; the direction is not in doubt.
Growth performance (BW/BWG/ADG)	Funnel plot and Egger's regression test	Egger's test: intercept =-0.31 , t=-0.24 , P=0.819 ( k=9 ). No evidence of small-study effects	No indication of publication bias, although the test has little interpretive value when the pooled effect is itself null. Harmonised reporting of growth outcomes remains needed.

The table reports the analyses used to assess the robustness of the findings and the possibility of small-study effects. For colonisation, it summarises the leave-one-out analysis, in which the sequential omission of each comparison preserved the negative direction of the pooled mean difference in every iteration but its statistical significance in five of six. It also gives the funnel-plot impression and the numerical result of Egger's regression test for both outcomes. Publication bias was assessed by visual inspection of the funnel plot (Fig. 6) and by Egger's test; no adjustment method was applied, because such methods are unreliable with fewer than 10 comparisons (Sterne et al., 2011).

For growth performance, Egger's test gave no indication of small-study effects (intercept =-0.31 , t=-0.24 , P=0.819 ). Because the pooled effect for this outcome was itself null, however, this result carries little interpretive weight. The overall position is therefore that the direction of the antimicrobial effect is stable, that its magnitude may be overestimated to an extent that cannot be quantified with the available number of studies and that no comparable concern arises for the growth outcome.

## 4 Discussion

The present systematic review and meta-analysis have brought together the available in vivo evidence on caprylic acid supplementation in broiler chickens, providing an updated quantitative estimate of its impact on *C. jejuni* colonisation and growth performance. The most consistent and practically relevant observation is that caprylic acid reduces caecal *C. jejuni* loads when it is administered in feed at an appropriate dose (Solís de los Santos et al., 2008, 2009, 2010). Within the in-feed subgroup, the reduction averaged 2.68 log10 CFU g^−1^. Quantitative risk assessment indicates that a 2-log reduction in carcass contamination would reduce the incidence of human campylobacteriosis associated with chicken meals roughly 30-fold (Rosenquist et al., 2003), which is why the magnitude matters as much as the direction. The present findings therefore support a role for caprylic acid within integrated Campylobacter control strategies, with the important qualification that the pooled estimate across all routes ( -1.46 log10 CFU g^−1^) understates the in-feed effect and overstates the effect of the compound as a class.

### 4.1 Sources of heterogeneity

Both outcomes were statistically heterogeneous ( $I^{2}=88.1$ % for colonisation and 72.4 % for growth), and locating the sources of that heterogeneity is more informative than the pooled averages themselves. For caecal colonisation, the dominant source was the delivery matrix. Stratifying by route reduced $I^{2}$ from 88.1 % overall to 39.0 % within the in-feed subgroup, and the test for subgroup differences was unambiguous ( Q _between = 14.57, df = 1, P<0.001 ); in other words, in-feed and water-based applications should not be regarded as estimating the same underlying quantity. A plausible mechanism is that feed provides a lipophilic, slow-release matrix in which undissociated caprylic acid survives the upper gastrointestinal tract, whereas aqueous formulations are rapidly absorbed by the enterocytes before reaching the ceca, where *C. jejuni* colonises the mucus layer and is shielded from fatty acid activity (Van Deun et al., 2008; Hermans et al., 2010). For growth performance, the corresponding partition was by formulation and dose: heterogeneity was entirely absent within the low-to-moderate-dose pure caprylic acid subgroup ( $I^{2}=0.0$ %, five comparisons), and the residual inconsistency in the overall estimate was generated by the two extremes of the dose–formulation range, namely a single high-dose (1.4 %) trial showing growth depression and blend-based interventions showing improvement ( Q _between = 16.61, df = 3, P<0.001 ).

Several further sources of variation could be identified qualitatively but could not be modelled quantitatively, because the number of comparisons per stratum was too small for meta-regression. These include the age of the birds at intervention, which ranged from 10 d old chicks to market-aged 42 d old broilers; the duration of supplementation, which ranged from 3 d to continuous administration throughout the production cycle; the presence or absence of a 12 h feed-withdrawal period before slaughter, which altered the measured effect within a single study (Solís de los Santos et al., 2009, 2010); the challenge model, since the growth studies included birds challenged with *Salmonella enteritidis* rather than *C. jejuni* (Kollanoor Johny et al., 2009; Kollanoor-Johny et al., 2012); and the experimental unit, which was the individual bird in some trials and the pen or cage in others. Residual heterogeneity of this kind is expected in a literature of this size and should be viewed as a limitation of the evidence base rather than of the synthesis, and as a specific argument for harmonised reporting in future trials.

This interpretation is consistent with experimental observations that micro-encapsulation prolongs the presence of fatty acids in the lower gut, and protected formulations therefore deserve direct examination in Campylobacter control (Timbermont et al., 2010; Gracia et al., 2016). It is also consistent with the negative dose–response result in drinking water: if the limiting step were the quantity of compound reaching the ceca rather than its survival en route, raising the water-borne concentration 32-fold should have produced some graded response, and it did not (Metcalf et al., 2011).

### 4.2 Dose, formulation and growth responses

The dose–response evidence sharpens what can be said about practical inclusion rates. Two features of the in-feed series are notable. The first is that the relationship is not monotonic: reductions of -2.2 to -3.6 log10 CFU g^−1^ were obtained consistently between 0.35 % and 0.875 %, whereas concentrations of 1.05 % and above produced weaker average reductions and, at 1.4 %, results that differed by nearly 4 log units between replicate trials of the same experiment. An intervention whose effect becomes less reproducible as the dose rises is not one whose dose should be raised. The second is that the upper limit is set by palatability rather than by pharmacology: feed intake fell steadily across the dose series and body weight was significantly depressed from 1.225 %, while concentrations up to 0.875 % left growth unaffected. The two outcomes examined in this review therefore converge on the same recommendation, which is the practical value of treating them as facets of a single product-quality objective: the interval between roughly 0.35 % and 0.875 % of feed delivers the antimicrobial benefit without a measurable zootechnical cost, and there is at present no empirical basis for exceeding it.

Two caveats attach to this conclusion. First, reduced feed intake does not by itself account for the attenuation of efficacy at high concentrations, as the total quantity of caprylic acid ingested continued to rise with dose; a saturable mechanism, or an adaptive response of the caecal microbiota, may be involved, and neither can be tested with the available data. Second, the entire dose–response description rests on two studies from two laboratories, one delivering the compound in feed and one in water, so it should be read as a hypothesis-generating summary rather than as an established exposure–response curve. A properly powered dose-titration trial with harmonised outcome measurement remains the obvious next step and would also permit the meta-regression that the present evidence base cannot support.

Caprylic acid has a dual character with respect to growth performance. Neutral effects at moderate doses mean that the compound can be used for pathogen reduction without a productivity penalty, which is what makes it economically viable (Kollanoor-Johny et al., 2012). The growth depression at 1.4 % of feed ( g=-1.89 ; 95 % CI -3.10 to -0.67 ) marks the upper safety limit, beyond which intake and body weight fall (Solís de los Santos et al., 2008). The apparently positive responses to blends require more caution than the previous literature has generally shown. In the two comparisons concerned, the caprylic acid dose was either negligible and confounded with a second bioactive compound (100 mg kg^−1^ with *Yucca schidigera* extract; Begum et al., 2015) or the compound formed one component of a monoglyceride mixture whose pairwise contrast with the control was not significant by the authors' own post-hoc test (Lin et al., 2022). Blends containing medium-chain fatty acids may well improve gut morphology, modulate the microbiota or enhance nutrient digestibility (Timbermont et al., 2010; Lin et al., 2022), but the present evidence does not attribute these benefits to caprylic acid specifically. The defensible statement is that caprylic acid is growth neutral within its effective antimicrobial range, not that it promotes growth when blended.

### 4.3 Robustness of the evidence and practical implications

The publication-bias analyses deserve a more careful reading than a significant Egger test alone would invite. Funnel-plot asymmetry has at least four possible origins: the selective publication of favourable results, poorer methodological quality among smaller trials, genuine clinical heterogeneity between large and small studies and chance (Sterne et al., 2011). In the present dataset, the third explanation is not merely plausible but demonstrable, because the asymmetry coincides exactly with the route-of-administration subgroup that also accounts for most of the statistical heterogeneity: the imprecise, strongly negative comparisons are the in-feed trials; and the precise, near-null comparisons are the drinking-water trials. Attributing the asymmetry wholly to publication bias would therefore double-count an effect we have already explained mechanistically. At the same time, selective publication cannot be excluded, and the small number of eligible comparisons ( k=6 ) means that no statistical procedure available to us can adjudicate between these explanations; trim and fill, and related adjustments are not recommended below 10 studies. The honest conclusion is that the pooled magnitude of -1.46 log10 CFU g^−1^ should be regarded as an upper bound on the true average in-feed effect rather than as an unbiased point estimate. The leave-one-out analysis nevertheless shows that the direction of the antimicrobial effect does not depend on any single comparison, even though its statistical significance is marginally dependent on one trial. The heterogeneity that remains after subgrouping is a reminder that unmeasured factors – broiler strain, basal diet composition, farm hygiene and the particular *C. jejuni* challenge strain – also contribute, and that they limit the precision with which any effect size can be generalised to commercial conditions (Hermans et al., 2011; Sahin et al., 2015).

These considerations have practical implications for both research and industry. From an industry viewpoint, the present evidence supports the use of caprylic acid by broiler integrations seeking to reduce Campylobacter risk without antibiotics, provided it is delivered in feed at approximately 0.35 % to 0.875 %, and preferably in micro-encapsulated or otherwise protected form. It does not support water-borne application, and it gives no reason to exceed 0.875 % (Timbermont et al., 2010; Gracia et al., 2016). From a research perspective, there is a clear need for more standardised and well-reported trials that systematically vary caprylic acid dose, formulation and timing of administration, ideally with harmonised outcome measures and detailed descriptions of husbandry conditions. Dose–response and meta-regression approaches could then be used more rigorously to identify truly optimal inclusion rates and regimens. Likewise, future work might investigate whether caprylic acid exerts additional benefits when combined with other non-antibiotic interventions – such as competitive exclusion cultures, bacteriophages or vaccination – in multifaceted control programmes (Meunier et al., 2016).

## 5 Conclusion

This systematic review and meta-analysis show that caprylic acid reduces caecal *Campylobacter jejuni* colonisation in broilers when it is delivered in feed by an average of 2.68 log10 CFU g^−1^ and that it does not do so when delivered in drinking water. Within its effective range of approximately 0.35 % to 0.875 % of feed, the compound is growth neutral; at 1.4 %, it depresses feed intake and body weight without conferring any additional antimicrobial benefit (Solís de los Santos et al., 2008). Subgroup, dose–response, sensitivity and publication-bias analyses confirm that the direction of the antimicrobial effect is robust, while showing that its magnitude is uncertain: substantial heterogeneity persists, funnel-plot asymmetry is significant and the pooled estimate should be read as an upper bound. Overall, caprylic acid has emerged as a promising element of antibiotic-free strategies aiming to mitigate Campylobacter in broiler production, but its practical implementation will require careful attention to dose, formulation and delivery route if gains in microbiological safety are to be achieved without sacrificing the technological quality attributes of the carcass.

## Supplement

10.5194/aab-69-477-2026-supplement The supplement related to this article is available online at https://doi.org/10.5194/aab-69-477-2026-supplement.

## Data Availability

All data extracted from the primary publications are compiled in tabular form and are available from the corresponding author on reasonable request. The study generated no unpublished primary data.

## Appendix A Studies included in the meta-analysis

**Table A1:** Twelve studies met the eligibility criteria. Eleven contributed 15 comparisons to the quantitative syntheses: six to the colonisation analysis and nine to the growth analysis. Hovorková and Skřivanová (2015) reported no numerical caecal counts and is discussed narratively only. van Gerwe et al. (2010) reported colonisation as a CD50 threshold, which is not commensurate with the log10 CFU g^−1^ scale and therefore contributes to the growth analysis alone. The number of comparisons contributed by each study is given in parentheses.

No.	Study	Contribution to the syntheses
1	Solís de los Santos et al. (2008)	Colonisation (1) and growth (2)
2	Solís de los Santos et al. (2009)	Colonisation (1); growth data not shown
3	Solís de los Santos et al. (2010)	Colonisation (1) and growth (1)
4	Metcalf et al. (2011)	Colonisation (2 replicate trials)
5	Hermans et al. (2012)	Colonisation (1)
6	van Gerwe et al. (2010)	Growth (1); colonisation reported as CD50, not poolable
7	Hovorková and Skřivanová (2015)	None; no numerical caecal data reported
8	Kollanoor Johny et al. (2009)	Growth (1)
9	Kollanoor-Johny et al. (2012)	Growth (1)
10	Begum et al. (2015)	Growth (1)
11	Lin et al. (2022)	Growth (1)
12	Hejdysz et al. (2012)	Growth (1)

All 12 references were verified against the primary publications. DOIs are given where assigned; Kollanoor Johny et al. (2009) predates DOI assignment in the *Journal of Food Protection*, and Hejdysz et al. (2012) appeared in an open-access national journal that does not assign DOIs.

## Code availability

No custom software package was developed for this study. All analyses were carried out with the script described in Sect. 2.5, which is available from the corresponding author on request.
