# Supplementary material for: Dietary caprylic acid, Campylobacter jejuni colonisation and product quality in broilers: a systematic review and meta-analysis
Source: Arch Anim Breed. 2026 Sep 2;69(3):477–93. doi: 10.5194/aab-69-477-2026 (PMC13555093; doi:10.5194/aab-69-477-2026)
Supplement: Supplementary file 1 [file aab-69-477-2026-supplement.zip › aab-69-477-2026-supplement-title-page.pdf]

Supplement of Arch. Anim. Breed., 69, 477–493, 2026
https://doi.org/10.5194/aab-69-477-2026-supplement
© Author(s) 2026. CC BY 4.0 License.

Open Access
Archives Animal Breeding

Supplement of

Dietary caprylic acid, *Campylobacter jejuni* colonisation and product quality in broilers: a systematic review and meta-analysis

Muhammet Kuddusi Erhan

Correspondence to: Muhammet Kuddusi Erhan (mkuddus@hotmail.com)

- aab-69-477-2026-supplement-title-page.pdf
- supplement
  - README.txt
  - S1_extracted_dataset.xlsx
  - S2_meta_analysis_figures1-6.py
  - S3_dose_response_figure7.py

The copyright of individual parts of the supplement might differ from the article licence.
